# Effects of *Clostridium butyricum* on antioxidant properties, meat quality and fatty acid composition of broiler birds

**DOI:** 10.1186/s12944-015-0035-0

**Published:** 2015-04-22

**Authors:** Xiudong Liao, Rujuan Wu, Guang Ma, Longmei Zhao, Zhaojun Zheng, Rijun Zhang

**Affiliations:** Laboratory of Feed Biotechnology, State Key Laboratory of Animal Nutrition, College of Animal Science and Technology, China Agricultural University, Beijing, 100193 People’s Republic of China

**Keywords:** *Clostridium butyricum*, Antioxidant properties, Meat quality, Fatty acid

## Abstract

**Background:**

Consumers are becoming increasingly interested in food containing high concentration of polyunsaturated fatty acids (PUFA). PUFA are considered as functional ingredients to prevent cardiovascular disease. The present study aimed to evaluate the effects of *Clostridium butyricum* on antioxidant properties, meat quality and fatty acid composition of broilers.

**Methods:**

A total of 320 one-day-old Arbor Acres male chicks were randomly assigned to one of five treatments with eight replicates and fed a antibiotic-free basal corn-soybean meal diet (control) or the basal diet supplemented with either 2.5 × 10^8^ (CB1), 5 × 10^8^ (CB2) or 1 × 10^9^ (CB3) cfu of *C. butyricum*/kg or150 mg of aureomycin/kg (antibiotic) for 42 days.

**Results:**

The results showed that chicks fed diets supplemented with *C. butyricum* had higher (*P* < 0.05) superoxide dismutase activity and lower (*P* < 0.05) malondialdehyde concentration in liver compared with those in the control group. Broilers had lower (*P* < 0.05) cholesterol content of serum in either CB2 or CB3 treatment at day 21 and in the *C. butyricum*-supplemented groups at day 42 than those in the control group. Chicks fed CB3 diet had lower (*P* < 0.05) percentage of abdominal fat and higher (*P* < 0.05) breast muscle yield than those in the control and antibiotic groups. The supplementation of *C. butyricum* increased (*P* < 0.05) the concentrations of C20:1n-9, C20:2n-6, C20:3n-6, C20:3n-3, C20:4n-6, C20:5n-3, C22:6n-3 and total PUFA as well as ratio of PUFA to saturated fatty acids in breast muscle and the contents of C18:2 *t*-9, *t*-12, C20:3n-6, C20:3n-3 and C20:5n-3 in thigh muscle.

**Conclusions:**

Supplementation of *C. butyricum* promotes hepatic antioxidant status, decreases cholesterol content of serum and percentage of abdominal fat, and improves meat quality and fatty acid composition of broiler birds. The results from the present study indicate that the increased PUFA concentrations in meat of broilers fed *C. butyricum* might be attributable to enhanced antioxidant activity.

## Background

The alteration of animal lipids has long been studied but still receives a lot of attention in research because of its implications for human health [1,2]. Furthermore, consumers are becoming increasingly interested in food containing high amount of polyunsaturated fatty acids (PUFA). PUFA are considered as functional ingredients to prevent cardiovascular disease and other chronic diseases [3,4]. Chicken meat is considered one of the most desirable meats all over the world as a result of relatively low fat content and high concentration of PUFA [5]. Moreover, meat quality and fatty acid composition of poultry can be easily modified by dietary mean [6,7]. Some studies indicated that dietary supplementation of probiotics (*Lactobacillus* and *Rhodobacter capsulatus*) increased PUFA concentration and reduced cholesterol level in chickens [8-11]. However, unsaturated fatty acids (UFA) are more sensitive to oxidation than saturated fatty acids (SFA) and particularly vulnerable to peroxidative attack [12]. Several studies have suggested that increased PUFA of meat could be the result of protective effect of dietary antioxidants, since these antioxidants act as electron donors to provide electrons for reduction of some UFA [5,13,14].

During the past decade, some studies have supported the potential reduction of oxidative stress of probiotics, such as *Lactobacillus* and *Bifidobacterium* [15-18]. Nevertheless, *Clostridium butyricum* can produce endospores and short-chain fatty acids, which has the ability to survive at lower pH, relatively higher bile concentrations and temperature compared with *Lactobacillus* and *Bifidobacterium* and has been used in a wide range of human and veterinary intestinal diseases as one important symbiotic bacteria [19-22]. To our knowledge, information is lacking on the effects of *C. butyricum* on antioxidant properties and fatty acid composition of broilers. It is now hypothesized that *C. butyricum* might increase PUFA concentration of meat through improving antioxidant activity in broiler chicks. Therefore, the objectives of this study were to assess the effects of *C. butyric*um on antioxidant properties, meat quality and fatty acid composition of broiler chicks.

## Methods

### Chicks, diets, and experimental design

All experimental procedures were approved by the China Agricultural University Animal Care and Use Committee (Beijing, China). A total of 320 one-day-old Arbor Acres male broilers (Huadu Broiler Breeding Corporation, Beijing, China) were randomly allotted to one of five treatments of eight replicate cages with eight birds per cage in a completely randomized design for 42 days. Chicks were housed in a electrically heated, thermostatically controlled room with feeders, nipple drinker and steel cages, and standard conditions of temperature, humidity, and ventilation were provided for the entire experimental period. Feed and tap water were available *ad libitum*. The basal corn-soybean meal diets were formulated to meet or exceed the National Research Council (1994) [23] requirements of broilers for all nutrients (Table 1). Dietary treatments were as follows: basal diet (control), the basal diet supplemented with 2.5 × 10^8^ cfu of *C. butyricum*/kg (CB1), 5 × 10^8^ cfu of *C. butyricum*/kg (CB2), 1 × 10^9^ cfu of *C. butyricum*/kg (CB3), and 150 mg of aureomycin/kg (antibiotic). The strain of *C. butyricum* used in this study was *C. butyricum* CGMCC 8187 (China General Microbiological Culture Collection Center, Beijing, China).

**Table 1:** **Composition of the basal diets for broilers (as-fed basis)**

**Item (% unless noted)**	**Starter (1 to 21 days)**	**Grower (22 to 42 days)**
Ingredient		
Corn	53.33	60.87
Soybean meal	38.75	32.04
Soy oil	3.70	3.26
Calcium monohydrogen phosphate	1.98	1.69
Ground limestone	1.05	1.08
Salt	0.35	0.35
DL-Met	0.18	0.12
Lysine	0.04	0.02
Choline chloride (50%)	0.30	0.25
Mineral premix^a^	0.30	0.30
Vitamin premix^b^	0.02	0.02
Calculated nutrient compositon		
ME, Mcal/kg	2.95	3.00
CP	21.00	19.00
Lysine	1.15	1.00
Methionine	0.50	0.40
Calcium	0.98	0.90
Nonphytate phosphorus	0.45	0.40

^a^Supplied per kilogram of diet: Mn, 100 mg; Fe, 80 mg; Zn, 75 mg; Cu, 8 mg; Se, 0.15 mg; I, 0.35 mg.

^b^Supplied per kilogram of diet: vitamin A, 12500 IU; cholecalciferol, 2500 IU; vitamin E, 30 IU; vitamin K_3_, 2.65 mg; thiamin, 2 mg; riboflavin, 6 mg; pantothenic acid, 12 mg; vitamin B_12_, 0.025 mg; niacin, 50 mg; biotin, 0.0325 mg; folic acid, 1.25 mg.

### Sample collections and analysis

At 21 days of age, only blood samples were taken from the wing vein of eight birds (one bird per cage) of each treatment and centrifuged at 3600 × g for 10 min at 4°C, serum was collected and stored at −20°C until analysis. At 42 days of age, eight chicks (one bird from each replicate cage) were weighed and chosen from each treatment based on average body weight within the pen following a 12-h fast. Blood samples were taken in the same way of the above-mentioned method. Then the birds were slaughtered and scalded in a hot water bath (60°C for 45 s) and the feathers removed mechanically after bleeding. The weight of carcass was measured after defeathering to determine the dressing percentage. Then abdominal fat (leaf fat surrounding the cloaca and abdominal fat surrounding the gizzard) was collected and weighed. The heads, feet, and organs, except the lungs and kidneys were removed and weighted to determine the percentage of eviscerated yield. The breast and thigh muscles were removed and weighed to determine the percentages of breast and leg muscle. They were then divided into two parts respectively: one was quickly frozen at −20°C for later determination of fatty acid composition, and another was used to measure meat color and pH value. Dressing, eviscerated yield and abdominal fat percentages were calculated by dividing these traits by final live weight after fasting. The percentages of breast muscle and thigh muscle were calculated as a percentage of eviscerated carcass weight.

### Meat quality

Meat color was measured on 3 points of every meat at 45 min after slaughter by a spectrocolorimeter (model WSC-S, Shanghai Shenguang Ltd., Shanghai, China) according to the CIE L*a*b* color system (where L* measures relative lightness, a* measures relative redness, and b* measures relative yellowness). The pH values of the breast and thigh muscles at a depth of 2.5 cm below the surface were measured at 45 min (pH_45min_, initial pH) and 24 h (pH_24h_, ultimate pH) postmortem using a Testo 205 pH meter (Testo AG, Lenzkirch, Germany) equipped with an insertion electrode. Three measurement values of pH_45min_ and pH_24h_ were recorded and averaged for each breast and thigh muscle.

### Assay of antioxidant indices

The activities of superoxide dismutase (SOD), glutathione S-transferase (GST) and glutathione peroxidase (GPX), concentrations of glutathione (GSH) and malondialdehyde (MDA) in liver and serum were measured using SOD, GST, GPX, GSH and MDA assay kits (Nanjing Jiancheng Bioengineering Institute, Nanjing, China) according to the manufacture’s protocols, respectively.

### Determination of serum biochemical parameters

Serum cholesterol, triglyceride (TG), high-density lipoprotein cholesterol (HDL-C), and low-density lipoprotein cholesterol (LDL-C) were measured with the commercial kits (Baiding Biological Engineering Co., Ltd., Beijing, China) and an automatic biochemistry analyzer TBA-120FR (Toshiba Medical Systems Co., Tokyo, Japan). All the procedures were carried out according to the manufacturers’ instructions.

### Fatty acid analysis

Fatty acid composition of breast and thigh muscles were determined by gas chromatography. The total lipids were extracted following the chloroform-methanol procedure of Folch et al. [24]. Total lipid extracts were transmethylated into fatty acid methyl esters using KOH in methanol and 14% methanolic boron triflouride and separated by using an HP 6890 gas chromatograph equipped with a flame-ionization detector and a DB-23 capillary column (internal diameter 0.25 mm, length 60 m, film thickness 0.25 μm; J&W Scientific, Folsom, CA, USA). Injector and detector temperatures were 250°C and 280°C, respectively. Nitrogen was used as the carrier gas at the flow rate of 1 mL/min. The oven temperature was 180°C held for 10 min, increased to 220°C at 4°C/min and held for 15 min, then to 250°C at 3°C/min and held for 30 min. The fatty acids were identified by comparison of their retention times with those of standards and concentrations were expressed as milligram per 1 g of muscle.

### Statistical analyses

Data from the experiment was analyzed by one-way ANOVA with the general liner model procedure of SAS (release 8.1, SAS Institute Inc., Cary, NC). Cage was the experimental unit. The *P* < 0.05 was considered to be statistically significant. Differences among means were tested by the least significant difference method.

## Results

### Antioxidant indices

Broilers fed either CB2 or CB3 diet had greater (*P* < 0.05) SOD activity in the liver than those in the control group (Table 2), but no differences (*P* < 0.05) in SOD activity were found among the control group, CB1 group and antibiotic group. However, the addition of *C. butyricum* significantly decreased (*P* < 0.05) MDA concentration in the liver compared with the control. No differences in GST and GPX activities, and GSH concentration in liver were detected (*P* > 0.05) among all the treatments.

**Table 2:** **Effect of** ***C. butyricum*** **on antioxidant indices in liver and serum of broilers**

**Item**	**Control**	**CB1**	**CB2**	**CB3**	**Antibiotic**	**Pooled SE**	***P*** **-value**
Liver^a^							
SOD (U/mg protein)	195c	212bc	240a	226ab	210bc	6	0.0002
GST (U/mg protein)	59.0	52.8	52.4	60.7	53.1	3.9	0.4546
GPX (U/mg protein)	8.49	9.45	9.19	8.67	9.26	0.36	0.2836
GSH (μmol/g protein)	10.09	9.39	10.32	9.39	9.36	0.50	0.5412
MDA (nmol/mg protein)	1.02a	0.88bc	0.82c	0.86bc	0.93ab	0.03	0.0014
Serum^a^							
SOD (U/ml)	118	110	128	113	107	9	0.5090
GST (U/ml)	22.9	22.7	22.4	21.5	19.8	0.8	0.0680
GPX (U)	785	797	786	867	890	46	0.4621
GSH (μmol/L)	29.9bc	32.5ab	38.0a	24.6 cd	22.1d	2.7	0.0016
MDA (nmol/ml)	2.36bc	2.37bc	2.24c	2.80ab	2.87a	0.16	0.0282

Means with different letters within the same row differ (*P* < 0.05).

Control = basal diet; CB1 = the basal diet supplemented with 2.5 × 10^8^ cfu of *C. butyricum*/kg; CB2 = the basal diet supplemented with 5 × 10^8^ cfu of *C. butyricum*/kg; CB3 = the basal diet supplemented with 1 × 10^9^ cfu of *C. butyricum*/kg; Antibiotic = the basal diet supplemented with 150 mg of aureomycin/kg.

SOD = superoxide dismutase; GST = glutathione S-transferase; GPX = glutathione peroxidase; GSH = glutathione; MDA = malondialdehyde.

^a^Each value represents the mean of 8 cages with one chick per cage (n = 8 per treatment).

The GSH concentration of serum was higher (*P* < 0.05) in the chicks fed both CB1 and CB2 diets in comparison with those fed the either CB3 or antibiotic diet (Table 2), and birds fed the CB2 diet had higher (*P* < 0.05) GSH content than those in the control group. Nevertheless, birds fed the control, CB1 and CB2 diets had lower (*P* < 0.05) MDA concentration of serum than those in the antibiotic group. No significant differences (*P* > 0.05) were observed in SOD, GST or GPX activities in serum among all the treatments.

### Serum biochemical parameters

Both cholesterol and HDL-C concentrations of serum at 21 days of age were lower (*P* < 0.05) in the chicks fed CB2 and CB3 diets compared with those fed the control diet (Table 3), but no differences (*P* > 0.05) were found in TG and LDL-C contents of serum at 21 days of age among the 5 treatments. There were no differences in TG, HDL-C and LDL-C contents were detected (*P* > 0.05) among all the treatments, but broilers in the *C. butyricum*-supplemented groups had lower (*P* < 0.05) cholesterol concentration at 42 days of age than those in the control group.

**Table 3:** **Effect of** ***C. butyricum*** **on serum biochemical parameters of broilers**

**Item**	**Control**	**CB1**	**CB2**	**CB3**	**Antibiotic**	**Pooled SE**	***P*** **-value**
21 days^a^							
Total cholesterol (mM)	3.78a	3.51abc	3.25c	3.42bc	3.65ab	0.1	0.0111
Triglyceride (mM)	0.74	0.72	0.75	0.68	0.67	0.10	0.0111
High-density lipoprotein cholesterol (mM)	2.35a	2.28ab	2.05c	2.10bc	2.23abc	0.07	0.0233
Low-density lipoprotein cholesterol (mM)	1.27	1.24	1.23	1.20	1.27	0.05	0.8976
42 days^a^							
Total cholesterol (mM)	3.37a	3.05b	2.96b	2.90b	3.09ab	0.1	0.0287
Triglyceride (mM)	0.41	0.37	0.30	0.32	0.36	0.03	0.1437
High-density lipoprotein cholesterol (mM)	2.06	1.93	1.81	1.88	2.02	0.07	0.1216
Low-density lipoprotein cholesterol (mM)	1.06	1.01	0.98	1.04	1.05	0.06	0.8519

Means with different letters within the same row differ (*P* < 0.05).

Control = basal diet; CB1 = the basal diet supplemented with 2.5 × 10^8^ cfu of *C. butyricum*/kg; CB2 = the basal diet supplemented with 5 × 10^8^ cfu of *C. butyricum*/kg; CB3 = the basal diet supplemented with 1 × 10^9^ cfu of *C. butyricum*/kg; Antibiotic = the basal diet supplemented with 150 mg of aureomycin/kg.

^a^Each value represents the mean of 8 cages with one chick per cage (n = 8 per treatment).

### Carcass traits

Broilers in the *C. butyricum*-supplemented groups had lower (*P* < 0.05) percentage of abdominal fat than those in the antibiotic group (Table 4); furthermore, broilers in the either CB1 or CB3 group had lower (*P* < 0.05) percentage of abdominal fat than those in the control group. However, broilers fed CB3 diet had higher (*P* < 0.05) breast muscle percentage than those in the control, CB1 and antibiotic groups. No significant differences (*P* > 0.05) were observed in percentages of dressing, eviscerated yield and thigh muscle among all the treatments.

**Table 4:** **Effect of** ***C. butyricum*** **on carcass traits of broilers**

**Item** ^**a,b**^	**Control**	**CB1**	**CB2**	**CB3**	**Antibiotic**	**Pooled SE**	***P*** **-value**
Dressing (%)	90.5	89.3	90.0	89.7	88.8	0.4	0.0764
Abdominal fat (%)	1.50ab	1.23c	1.30bc	1.27c	1.57a	0.08	0.0339
Eviscerated yield (%)	71.2	71.5	71.8	72.1	72.0	0.6	0.8087
Breast muscle (%)	26.8b	27.8b	28.0ab	29.9a	27.5 b	0.7	0.0367
Thigh muscle (%)	22.5	22.0	21.8	22.3	21.2	0.5	0.4417

Means with different letters within the same row differ (*P* < 0.05).

Control = basal diet; CB1 = the basal diet supplemented with 2.5 × 10^8^ cfu of *C. butyricum*/kg; CB2 = the basal diet supplemented with 5 × 10^8^ cfu of *C. butyricum*/kg; CB3 = the basal diet supplemented with 1 × 10^9^ cfu of *C. butyricum*/kg; Antibiotic = the basal diet supplemented with 150 mg of aureomycin/kg.

^a^Each value represents the mean of 8 cages with one chick per cage (n = 8 per treatment).

^b^Dressing, abdominal fat and eviscerated yield percentages were calculated by dividing these traits by final live weight after fasting. The percentages of breast muscle and thigh muscle were calculated as a percentage of eviscerated carcass weight.

### Fatty acid composition

There were significant differences (*P* < 0.05) in some fatty acid concentrations in the breast muscle amongst the treatments (Table 5). For SFA, broilers fed CB2 diet resulted in higher (*P* < 0.05) concentration of stearic acid (C18:0) than those in the control, CB1 and antibiotic groups, but no significant differences (*P* > 0.05) were observed in other SFA and total SFA contents among all the treatments. For monounsaturated fatty acids (MUFA), broilers fed either CB1or CB2 diet had higher (*P* < 0.05) concentration of gondoic acid (C20:1n-9) than those in the control group. Among PUFA, the addition of *C. butyricum* significantly affected (*P* < 0.05) the concentrations of eicosadienoic acid (C20:2n-6), dihomo-gamma-linolenic acid (DGLA: C20:3n-6), eicosatrienoic acid (ETE: C20:3n-3), arachidonic acid (AA: C20:4n-6), eicosapentaenoic acid (EPA: C20:5n-3), docosahexaenoic acid (DHA: C22:6n-3), total PUFA and ratio of PUFA to SFA. Chicks fed CB2, CB3 and antibiotic diets had greater (*P* < 0.05) AA concentration than those in the control group. Birds fed either CB1 or CB2 diet had higher (*P* < 0.05) EPA concentration than those in the control group. In comparison with the control group, the *C. butyricum*-supplemented and antibiotic groups had higher (*P* < 0.05) DHA concentration. Moreover, birds in the CB2, CB3 and antibiotic groups had higher (*P* < 0.05) total PUFA concentration than those in the control group, and birds in the *C. butyricum*-supplemented and antibiotic groups had higher (*P* < 0.05) ratio of PUFA to SFA than those in the control group. Nevertheless, no significant differences (*P* > 0.05) were observed in other UFA in breast muscle among all the treatments.

**Table 5:** **Effect of** ***C. butyricum*** **on fatty acid contents (mg/g of dried meat) of breast meat**

**Item** ^**a**^	**Control**	**CB1**	**CB2**	**CB3**	**Antibiotic**	**Pooled SE**	***P*** **-value**
C14:0	0.155	0.157	0.165	0.189	0.168	0.018	0.6914
C15:0	0.020	0.027	0.016	0.034	0.018	0.006	0.2711
C16:0	8.18	9.03	10.68	9.34	9.31	0.85	0.3929
C17:0	0.041	0.049	0.048	0.065	0.041	0.006	0.0649
C18:0	3.66b	4.29b	5.39a	4.47ab	4.31b	0.32	0.0166
C20:0	0.064	0.132	0.106	0.085	0.083	0.020	0.2094
ΣSFA	12.12	13.69	16.41	14.18	13.93	1.18	0.1935
C14:1	0.017	0.016	0.004	0.016	0.015	0.007	0.6861
C16:1*c*	1.09	1.05	1.22	1.03	1.07	0.17	0.9427
C18:1 *t*	0.169	0.166	0.209	0.210	0.163	0.020	0.2673
C18:1*c*	9.01	10.50	13.31	11.39	10.76	1.35	0.3024
C20:1n-9	0.112c	0.190ab	0.209a	0.152abc	0.145bc	0.021	0.0277
C22:1n-9	0.018	0.051	0.042	0.043	0.047	0.009	0.1230
ΣMUFA	10.42	11.97	14.99	12.84	12.20	1.54	0.3710
C18:2 *t*-9, *t*-12	0.008	0.032	0.020	0.016	0.006	0.007	0.0917
C18:2*c*	7.94	9.81	12.77	10.69	10.64	1.05	0.0552
C18:3n-6	0.022	0.031	0.013	0.023	0.021	0.006	0.3856
C18:3n-3	0.456	0.606	0.769	0.676	0.693	0.089	0.1965
C20:2n-6	0.233b	0.374a	0.424a	0.327ab	0.341a	0.036	0.0106
C20:3n-6	0.070c	0.130b	0.195a	0.110b	0.100bc	0.011	<.0001
C20:3n-3	0.415c	0.573b	0.725a	0.512bc	0.540bc	0.048	0.0016
C20:4n-6	1.20c	1.80bc	3.35a	2.24b	2.22b	0.21	<.0001
C20:5n-3	0.100c	0.143ab	0.181a	0.123bc	0.123bc	0.014	0.0047
C22:6n-3	0.201b	0.341a	0.366a	0.301a	0.293a	0.026	0.0010
ΣPUFA	10.60c	13.86bc	18.04a	15.67ab	14.98ab	1.37	0.0103
PUFA/SFA	0.87d	1.01c	1.14a	1.07bc	1.08ab	0.02	<.0001

Means with different letters within the same row differ (*P* < 0.05).

Control = basal diet; CB1 = the basal diet supplemented with 2.5 × 10^8^ cfu of *C. butyricum*/kg; CB2 = the basal diet supplemented with 5 × 10^8^ cfu of *C. butyricum*/kg; CB3 = the basal diet supplemented with 1 × 10^9^ cfu of *C. butyricum*/kg; Antibiotic = the basal diet supplemented with 150 mg of aureomycin/kg.

*c* = *cis*; *t* = *trans*.

^a^Each value represents the mean of 8 cages with one chick per cage (n = 8 per treatment).

The *trans, trans*-linoleic acid (C18:2 *t*-9, *t*-12), DGLA (C20:3n-6), ETE (C20:3n-3) and EPA (C20:5n-3) contents in thigh muscle were significantly affected (*P* < 0.05) by dietary treatments (Table 6). Birds fed control and *C. butyricum*-supplemented diets had greater (*P* < 0.05) *trans, trans*-linoleic acid and DGLA concentrations than those in the antibiotic group, and birds fed control, CB1 and CB2 diets had greater (*P* < 0.05) ETE and EPA concentrations than those in the antibiotic group. However, no significant differences (*P* > 0.05) in other fatty acids, total SFA, total MUFA, total PUFA and the ratio of PUFA to SFA in the thigh muscle were detected (*P* > 0.05) among all the treatments.

**Table 6:** **Effect of** ***C. butyricum*** **on fatty acid contents (mg/g of dried meat) of thigh meat**

**Item** ^**a**^	**Control**	**CB1**	**CB2**	**CB3**	**Antibiotic**	**Pooled SE**	***P*** **-value**
C14:0	0.317	0.298	0.282	0.277	0.273	0.032	0.8660
C15:0	0.059	0.053	0.053	0.052	0.052	0.006	0.8735
C16:0	14.89	13.97	14.00	12.74	12.36	1.49	0.7518
C17:0	0.118	0.137	0.101	0.169	0.087	0.032	0.4256
C18:0	7.02	6.43	6.96	6.53	5.80	0.46	0.3515
C20:0	0.129	0.116	0.140	0.109	0.104	0.018	0.5969
ΣSFA	22.53	21.00	21.54	19.88	18.67	1.96	0.6780
C14:1	0.051	0.051	0.050	0.036	0.040	0.013	0.8877
C16:1*c*	2.43	2.20	2.17	1.77	1.87	0.36	0.7030
C17:1	0.027	0.025	0.022	0.017	0.007	0.005	0.1083
C18:1 *t*	0.230	0.200	0.223	0.194	0.321	0.045	0.2993
C18:1*c*	18.68	16.47	17.49	15.79	14.96	2.40	0.8291
C20:1n-9	0.233	0.200	0.264	0.250	0.173	0.027	0.1286
C22:1n-9	0.034	0.041	0.045	0.045	0.033	0.007	0.5814
ΣMUFA	21.68	19.18	20.27	18.10	17.40	2.80	0.8268
C18:2 *t*-9, *t*-12	0.035a	0.033a	0.046a	0.038a	0.010b	0.006	0.0034
C18:2*c*	16.41	15.85	17.28	15.76	14.99	1.81	0.9246
C18:3n-6	0.057	0.043	0.073	0.046	0.041	0.011	0.2616
C18:3n-3	0.990	0.993	1.078	0.960	0.982	0.153	0.9857
C20:2n-6	0.358	0.380	0.373	0.362	0.287	0.041	0.5251
C20:3n-6	0.131b	0.140ab	0.151ab	0.157a	0.101c	0.008	0.0003
C20:3n-3	0.592a	0.574a	0.636a	0.561ab	0.463b	0.036	0.0248
C20:4n-6	2.25	2.09	2.36	2.24	1.97	0.14	0.3483
C20:5n-3	0.128a	0.122a	0.136a	0.115ab	0.093b	0.009	0.0327
C22:6n-3	0.366	0.385	0.390	0.377	0.302	0.032	0.3040
ΣPUFA	21.32	20.61	22.53	20.61	19.24	2.13	0.8650
PUFA/SFA	0.95	0.98	1.04	1.02	1.04	0.03	0.1390

Means with different letters within the same row differ (*P* < 0.05).

Control = basal diet; CB1 = the basal diet supplemented with 2.5 × 10^8^ cfu of *C. butyricum*/kg; CB2 = the basal diet supplemented with 5 × 10^8^ cfu of *C. butyricum*/kg; CB3 = the basal diet supplemented with 1 × 10^9^ cfu of *C. butyricum*/kg; Antibiotic = the basal diet supplemented with 150 mg of aureomycin/kg.

*c* = *cis*; *t* = *trans.*

^a^Each value represents the mean of 8 cages with one chick per cage (n = 8 per treatment).

### Meat quality

Birds fed either CB1 or antibiotic diet had greater (*P* < 0.05) pH_45min_ value in the breast than those in the control or CB2 group (Table 7), whereas birds fed CB1, CB2 or antibiotic diet had greater (*P* < 0.05) pH_45min_ value in the thigh muscle than those in the control group. However, no differences (*P* > 0.05) in pH_24h_ values of breast and thigh muscles were detected among all the treatments.

**Table 7:** **Effect of** ***C. butyricum*** **on meat quality of broilers**

**Item**	**Control**	**CB1**	**CB2**	**CB3**	**Antibiotic**	**Pooled SE**	***P*** **-value**
Breast muscle^a^							
Lightness	56.8	54.0	57.5	59.4	55.3	1.6	0.1562
Redness	11.1	11.1	10.6	10.0	10.4	0.7	0.7995
Yellowness	12.2ab	11.1b	11.1b	12.8ab	13.9a	0.7	0.0240
pH_45min_	6.19b	6.49a	6.12b	6.27ab	6.48a	0.09	0.0119
pH_24h_	5.98	5.96	6.04	6.06	6.01	0.03	0.2033
Thigh muscle^a^							
Lightness	59.3	58.6	60.2	58.2	58.0	1.3	0.7344
Redness	14.0	14.5	12.4	13.1	13.0	0.6	0.1742
Yellowness	10.3	9.8	9.7	12.0	12.9	1.0	0.1027
pH_45min_	6.41c	6.60a	6.53ab	6.45bc	6.55ab	0.04	0.0049
pH_24h_	6.58	6.69	6.67	6.61	6.78	0.08	0.3774

Means with different letters within the same row differ (*P* < 0.05).

Control = basal diet; CB1 = the basal diet supplemented with 2.5 × 10^8^ cfu *C. butyricum*/kg; CB2 = the basal diet supplemented with 5 × 10^8^ cfu *C. butyricum*/kg; CB3 = the basal diet supplemented with 1 × 10^9^ cfu *C. butyricum*/kg; Antibiotic = the basal diet supplemented with 150 mg of aureomycin/kg.

pH_45min_ (45 min postmortem); pH_24h_ (24 h postmortem).

^a^Each value represents the mean of 8 cages with one chick per cage (n = 8 per treatment).

Broilers in the either CB1 or CB2 group had lower (*P* < 0.05) b* value of breast than those in the antibiotic group (Table 7). However, no significant differences (*P* > 0.05) in L* and a* values in the breast muscle and L*, a* and b* values in the thigh muscle were detected among all the treatments.

## Discussion

In recent years, consumers are becoming increasingly aware of the nutritional quality and healthy benefits of the food they consume. It is widely acknowledged that cholesterol content and fatty acid composition in poultry products are closely related to the occurrence of cardiovascular heart diseases [25]. Therefore, much attention has been paid toward the modulation of poultry products to decrease the risk of cardiovascular disease by improving the properties of carcasses and meat quality [5]. The present study showed that deposition of abdominal fat and serum cholesterol content in broiler chicks could be decreased while breast muscle yield and some PUFA concentrations of breast and thigh muscles could be increased by supplementing *C. butyricum* in the diet.

Some experiments were conducted to investigate the effects of probiotics on carcass traits and meat quality in animals. Suo et al. [26] found that *L. plantarum* ZJ316 had probiotic effects on improving pork quality, pH_45min_, hardness, stickiness, chewiness, gumminess and restoring force were all improved in *Lactobacillus*-treated pigs. Endo and Nakano [27] reported that dietary supplementation of probiotics (*Bacillus*, *Lactobacillus, Streptococcus*, *Clostridium*, *Saccharomyces*, and *Candida spp.*) enhanced the characteristics of carcass and meat quality in broilers. Results from the current study demonstrated that the addition of *C. butyricum* significantly influenced percentages of abdominal fat and breast muscle, pH_45min_ and b* values. Supplementation of *C. butyricum* resulted in better body composition with higher breast meat yield and lower abdominal fat percentage in broilers, which could be beneficial for poultry production.

The present data demonstrate that cholesterol and HDL-C contents of serum in broilers were decreased by *C. butyricum* supplementation at a level of either 5 × 10^8^ or 1 × 10^9^ cfu/kg of diet. Similar results were observed in the previous studies, indicating that supplemental *C. butyricum* decreased plasma and hepatic cholesterol levels and enhanced bile acid excretion in cholesterol-fed rats [28-30]. Furthermore, some studies have shown that *Lactobacillus* or *Bacillus* reduced cholesterol concentration in serum [31,32], in egg yolk [33,10], as well as in liver and carcass [9] of broiler chickens. The mechanisms for the cholesterol-lowering action of probiotic bacteria remains unclear, but it has been suggested that the effect may be attributed to binding of cholesterol to the bacterial cell wall, retarding cholesterol synthesis or increasing degradation of cholesterol by gut bacterial enzymes [9,34,35]. In addition, the development of adipose tissue depends on the availability of serum lipids which are principal substrates in lipid metabolism [36], suggesting that the decreased abdominal fat content evoked by *C. butyricum* supplementation as observed in this study might be associated with reduced serum lipids.

Fatty acid profile in animal products depends strongly on the fatty acid composition of the diet. Human nutritionists are recommending a higher intake of PUFA, which have a wide range of biological roles and are believed to be beneficial for human health [2,4]. In the present study, dietary supplementation of *C. butyricum* to broilers increased some PUFA contents and the ratio of PUFA to SFA in the breast and thigh muscles, especially for AA, EPA and DHA, which were supported by the study of Yang et al. [7], who reported that dietary *C. butyricum* increased AA and total n-3 fatty acid contents in breast meat of chickens. Moreover, the similar phenomenon was also reported in other studies [11,32], in which increased levels of AA and UFA/SFA ratio were found in the liver and meat of broilers supplemented with other probiotics (*Lactobacillus* and *Rhodobacter capsulatus*). These above results can be attractive to the consumers as high ratio of PUFA to SFA has a positive health benefit for humans, mainly in protection against cardiovascular disease [3].

The mechanisms by which dietary *C. butyricum* regulates PUFA contents of meat are still unclear. Notwithstanding, it is well recognized that probiotics can cause a positive effect on animal health through administering the digestive tract and affecting PUFA digestion and absorption processes in the gastrointestinal tract [7,37]. Recently, some studies have suggested that increased PUFA concentrations in meat could be on account of the protective role of dietary antioxidants [5,14,38]. Higher level of antioxidative enzymes will lead to reduction of the rate of lipid peroxidation, which means reduction of the rate of PUFA degradation by peroxidation [14]. Results from the present study showed that *C. butyricum* could inhibit both liver and blood lipid oxidation (MDA production) in broiler chicks, therefore protecting the peroxidation of oxidative-labile PUFA of meat. During the past decade, several studies have supported the potential reduction of oxidative stress of probiotics, such as *Lactobacillus* and *Bifidobacterium* [15-18]. The current study found that dietary supplementation of *C. butyricum* increased hepatic SOD activity and serum GSH content whereas decreased MDA concentration in both liver and serum of broilers. These results were supported by a recent study [39], which indicated that *C. butyricum*-treated rats showed remarkable induction of nuclear factor erythoid 2-related factor 2 (Nrf2) and its targeted antioxidative enzymes and suppressed hepatic oxidative stress. Moreover, *C. butyricum* can produce both butyric acid and H_2_ [40,41], the effects of butyrate on colonic mucosal health and antioxidative activity have been widely studied [42,43]; and recent studies have revealed that molecular H_2_ mediates beneficial effects in different systems as an optimal antioxidant agent by selectively scavenging free hydroxyl radicals (•OH) [44]. Thus, *C. butyricum* exert antioxidant properties may be partially attributed to the benefit effects of butyrate and H_2_ on reduction of oxidative stress. This could be supported by a previous study, which demonstrated that dietary sodium butyrate alleviated the oxidative stress induced by corticosterone exposure and increased AA contents in breast muscle of broilers [36]. Furthermore, other studies have indicated that some probiotic strains exert antioxidant activity and may be useful in reducing systemic oxidative stress through either stimulating the immune system reduces inflammation or increasing the amount of probiotics while inhibiting intestinal pathogens reduces inflammation and its associated oxidative injury [16,17,20,39]. However, the exact mechanisms need to be further studied.

## Conclusions

In summary, the results from the current study indicate that supplementation of *C. butyricum* promotes antioxidant status in liver and serum, decreases cholesterol and HDL-C concentrations in serum, and improves meat quality and fatty acid composition of broiler birds. Consumption of such a meat product may be beneficial to human health. The increased concentrations of PUFA observed in muscle of broilers fed a diet supplemented with *C. butyricum* may be as a result of the enhancement of antioxidant defenses in the host and deserves further investigation.
